# Identification of genes preferentially expressed in wild strawberry receptacle fruit and demonstration of their promoter activities

**DOI:** 10.1038/s41438-019-0134-6

**Published:** 2019-05-01

**Authors:** Rachel Shahan, Dongdong Li, Zhongchi Liu

**Affiliations:** 1 Department of Cell Biology and Molecular Genetics, University of Maryland, College Park, MD 20742 USA; 2 Zhejiang University, College of Biosystems Engineering and Food Science, Key Laboratory of Agro-Products Postharvest Handling Ministry of Agriculture, Zhejiang Key Laboratory for Agri-Food Processing, Zhejiang University, Hangzhou, 310058 People’s Republic of China; 3 Present Address: Department of Biology and Howard Hughes Medical Institute, Duke University, Durham, NC 27708 USA

**Keywords:** Model plants, Transcriptomics, Fruiting

## Abstract

*Fragaria vesca* (*F. vesca*), the wild strawberry, is a diploid model for the commercial, octoploid strawberry as well as other members of the economically relevant *Rosaceae* family. Unlike the fruits of tomato and *Arabidopsis*, the fleshy fruit of strawberry is unique in that it is derived from the floral receptacle and has an external seed configuration. Thus, identification and subsequent characterization of receptacle-expressed genes may shed light on novel developmental processes or provide insight into how developmental regulation differs between receptacle-derived and ovary-derived fruits. Further, since fruit and flower tissues are the last organs to form on a plant, the development of receptacle fruit-specific promoters may provide useful molecular tools for research and application. In this work, we mined previously generated RNA-Seq datasets and identified 589 genes preferentially expressed in the strawberry receptacle versus all other profiled tissues. Promoters of a select subset of the 589 genes were isolated and their activities tested using a GUS transcriptional reporter. These promoters may now be used by the *F. vesca* research community for a variety of purposes, including driving expression of tissue-specific reporters, RNAi constructs, or specific genes to manipulate fruit development. Further, identified genes with receptacle-specific expression patterns, including MADS-Box and KNOX family transcription factors, are potential key regulators of fleshy fruit development and attractive candidates for functional characterization.

## A fruitful investigation of strawberry production

A newly-acquired collection of gene regulatory elements gives researchers a powerful toolbox for studying fruit production in strawberry plants. Tissue-specific gene expression is governed by DNA sequences known as promoters, and researchers led by Rachel Shahan and Zhongchi Liu at the University of Maryland recently set out to identify promoters that regulate the initiation of strawberry formation. Most fruits form in the plant ovary, but strawberries originate from the same ‘receptacle’ structure that gives rise to flowers. Liu and Shahan therefore used RNA sequencing to identify genes that are expressed specifically in this tissue but not elsewhere in the strawberry plant. Their efforts uncovered seven promoters that appear to operate in a highly receptacle-specific fashion. Armed with these sequences, researchers can now begin to manipulate and thereby dissect the genetic mechanisms governing strawberry fruit formation.

## Introduction

*Fragaria vesca* (*F. vesca)*, the woodland strawberry, has been prized as an ornamental for centuries. *F. vesca* is an ancestor of *F. virginiana* which, when crossed with *F. chiloensis* in the eighteenth century, produced *F. ananassa*, the present-day commercial strawberry^1^. *F. vesca* is diploid (2n = 14), has a small and sequenced genome (240 Mb), is amenable to transformation, and has a short life cycle^2–4^. Comparative genetic mapping indicated a high degree of macrosynteny and colinearity between the genomes of *F. ananassa* and *F. vesca*, suggesting that molecular research conducted in the diploid *vesca* is likely applicable to the octoploid *ananassa*^5^. Unlike the ovary-derived fruits of *Arabidopsis* and tomato, the fleshy fruit of strawberry originates from the floral receptacle^6^. Comparison of ovary and receptacle-derived fruits is a powerful method to provide insight into general developmental processes.

Identification of genes involved in the spatial and temporal regulation of fruit development is useful for both fundamental developmental research and crop improvement. Strawberry is a valuable crop worldwide and much research has been focused on aspects of the latter stages of development such as fruit ripening, color, flavor, and nutrition^7–11^. However, fruit set, the point at which the decision to abort or initiate fruit development is made, is equally important for fruit production. Despite its relevance to crop yield, the molecular regulation of fruit set is not well understood. For example, although the homeotic genes regulating floral identity are characterized in detail^12^, an enduring question is the nature of the mechanism underlying promotion of fruit identity post fertilization.

To characterize the functions of genes of interest, such as candidate regulators of fruit set and fruit identity, established genetic methods include gene overexpression or knockdown/knockout. However, a common challenge with this approach is off-target effects caused by the use of broadly expressed promoters. Tissue-specific promoters are necessary to bypass the problems associated with broadly expressed promoters^13^ but, unlike *Arabidopsis*, few are currently available for strawberry. Previous work over the last two decades has endeavored to identify genes and regulatory sequences appropriate for driving gene expression in the fruit of the cultivated octoploid strawberry^14–19^. Initial efforts identified heterologous promoters from petunia that were functional in strawberry fruit^14^, though attention quickly turned to isolating genes and regulatory sequences from strawberry itself^15–19^. However, previous studies were conducted prior to publication of *F. vesca* genome and availability of genome-scale transcriptome studies of multiple tissues and developmental stages. Thus, fruit-specific genes were later found to have broader expression than previously realized.

In this study, we took advantage of genome-scale RNA-sequencing (RNA-Seq) data profiling multiple tissues and stages of *F. vesca* flower and fruit^20–22^ enabled by recent advancements in genome research. By conducting differential gene expression analysis, we identified genes more highly expressed in the developing receptacle than in any of the other profiled floral or vegetative tissues. From the list of receptacle-associated genes, we selected a subset of genes, isolated their upstream regulatory sequences, and demonstrated the sequences’ promoter activities in the developing receptacle via the β-glucuronidase (GUS) reporter. Due to the demonstrated similarities between the *F. vesca* and *F. ananassa* genomes^5^, the identified promoter sequences may also be applicable to research with cultivated strawberry. Further, the identified receptacle-expressed genes, especially MADS-Box transcription factors, are attractive candidates for future study and may provide insight into the unique development of strawberry’s fleshy fruit from the floral receptacle.

## Results

### Identification of receptacle fruit preferentially expressed genes using comprehensive RNA-Seq datasets

We mined extensive RNA-Seq datasets previously generated in *F. vesca* Yellow Wonder 5AF7 ^20–22^. These datasets consist of 92 RNA-Seq libraries representing 46 different tissues and stages (46 libraries × 2 biological replicates = 92; Table S1). Twenty-six libraries (13 libraries × 2 biological replicates = 26) represent receptacle tissues collected at multiple developmental timepoints ranging from young floral stages (floral stage 6 to 7), to the early stages of fruit development (fruit stages 1–5), and to the white (also called turning) stage*. F. vesca* fruit and flower staging has previously been described in detail^6,23^.

**Fig. 1:**
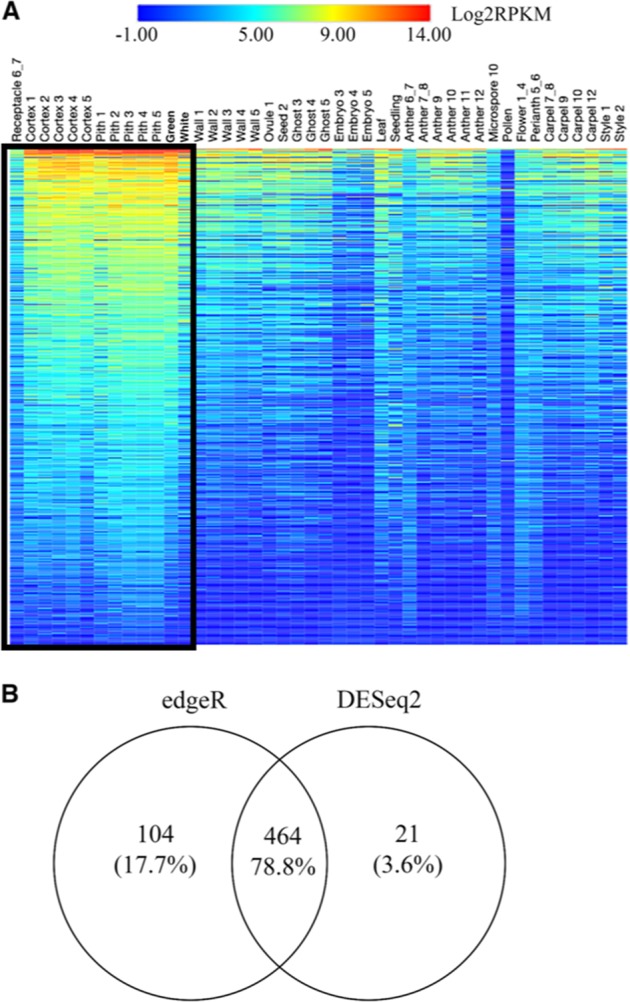
Differential expression analyses identified 589 receptacle preferentially expressed (RP) genes. **a** Heatmap showing expression profiles of 589 RP genes across all profiled tissues and stages of development (see Table S1 for description of each tissue). Each row represents one gene and each column represents a specific tissue. Receptacle tissues are outlined by the black box. Warmer colors denote higher expression. Log2 transformed reads per kilobase million (RPKM) values were used as input to generate the heatmap. RPKM values from the two biological replicates of each library were averaged. **b** Venn diagram indicating numbers of differentially expressed genes identified by the edgeR and DESeq2 packages

We asked what key genes may be involved in stimulating the floral receptacle to develop into a fleshy fruit. Differential expression analyses using both the DESeq2^24^ and edgeR^25^ packages identified genes that are more highly expressed in the receptacle than in any of the other profiled flower, fruit, or vegetative tissues (hereafter called ‘*r*eceptacle *p*referentially expressed (RP) genes’). In total, 589 RP genes were identified with at least a fourfold higher expression in the receptacle than in all other profiled tissues (Fig. 1a; Dataset S1). The majority, nearly 79%, of the 589 genes were identified by both edgeR and DESeq2 (Fig. 1b).

We asked what biological processes are overrepresented among the 589 RP genes, reasoning that key regulators of fruit development will be those that control these processes. Among the 16 categories of biological process Gene Ontology (GO) terms enriched in the list of RP genes, 3 involve response to abiotic stimuli, including light and radiation (Table 1). Ten categories are metabolic or biosynthetic processes, including regulation of nucleic acid metabolic process, regulation of RNA metabolic process, and regulation of macromolecule biosynthetic process. Three of the enriched GO terms involve transcriptional regulation of gene expression (Table 1). Factors modulating active transcription in the receptacle are potentially key regulators of fruit development. We found that 52 of the 589 RP genes are annotated as transcription factors (Dataset S2) and represented families include MYB, WRKY, KNOX, and MADS-Box. Of particular interest is the MADS-Box transcription factor family, the members of which have previously been implicated in floral identity specification^12^ and fruit ripening, e.g., *RIN*^26^. The RP genes annotated as MADS-Box transcription factors are gene06301 (*FveAGAMOUS-LIKE17*; *FveAGL17*), gene30741 (*FveSHORT VEGETATIVE PHASE*; *FveSVP*), gene26118 (*FveSEPALLATA1*; *FveSEP1*), and gene26119 (*FveFRUITFULL; FveFUL*). In *Arabidopsis, SEPALLATA1* is an E class gene that promotes floral organ identity^27^ and two homologs of *FRUITFULL* are involved in tomato fruit ripening^28^. Interestingly, *FveSEP1* and *FveFUL* are highly expressed throughout all profiled stages of receptacle fruit development (stage 1 to white) and are more highly expressed in the receptacle than in any profiled floral tissues (Dataset S1). This expression profile raises the possibility that *FveSEP1* and *FveFUL* function throughout receptacle fruit development and may not be limited to roles in floral organ identity and ripening.

**Table 1:** Biological process GO terms enriched among the 589 RP genes identified via differential expression analyses

GO term	Description	No. of genes out of 589 RP genes	No. of genes in reference genome	*P* value	FDR
GO:0009314	Response to radiation	6	34	0.0002	0.026
GO:0009416	Response to light stimulus	6	34	0.0002	0.026
GO:0009628	Response to abiotic stimulus	7	41	7.00E-05	0.026
GO:0019222	Regulation of metabolic process	38	1031	0.0016	0.046
GO:0031323	Regulation of cellular metabolic process	38	1018	0.0013	0.046
GO:0031326	Regulation of cellular biosynthetic process	37	995	0.0016	0.046
GO:0080090	Regulation of primary metabolic process	37	998	0.0017	0.046
GO:0019219	Regulation of nucleobase, nucleoside, and nucleic acid metabolic process	37	991	0.0015	0.046
GO:0009889	Regulation of biosynthetic process	37	995	0.0016	0.046
GO:0051171	Regulation of nitrogen compound metabolic process	37	991	0.0015	0.046
GO:0010468	Regulation of gene expression	37	1002	0.0018	0.046
GO:0051252	Regulation of RNA metabolic process	37	982	0.0013	0.046
GO:0006355	Regulation of transcription, DNA-dependent	37	982	0.0013	0.046
GO:0010556	Regulation of macromolecule biosynthetic process	37	995	0.0016	0.046
GO:0045449	Regulation of transcription	37	984	0.0013	0.046
GO:0060255	Regulation of macromolecule metabolic process	37	1009	0.002	0.049

*GO* Gene Ontology, *RP* receptacle preferentially expressed, *FDR* false discovery rate

### Selection and isolation of RP genes for the development of tissue-specific promoters

**Fig. 2:**
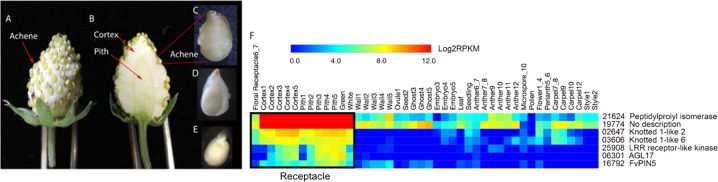
Expression profiles of seven receptacle preferentially expressed (RP) genes chosen for further characterization. **a** The botanical fruit of strawberry, the achenes, are externally arranged on the fleshy receptacle. **b** The fleshy receptacle is made up of two sub-tissues, the cortex and pith. Pith contains mostly vasculature. **c** The hard, outer shell of the achene is derived from the ovary wall. **d** Within an achene is a single seed, which houses an embryo (**e**)**. f** Heatmap showing expression profiles of 7 RP genes across all profiled tissues and stages of development. Black box indicates receptacle tissues. Numbers following the tissue names indicate developmental stage. Each row represents one gene. Gene descriptions were taken from Plaza 3.0. Warmer colors denote higher expression. Log2 transformed reads per kilobase million (RPKM) values were used as input to generate the heatmap. Average RPKM from the two biological replicates of each library are presented

Promoters of genes highly or specifically expressed in the receptacle fruit could serve as molecular tools to advance research in *F. vesca* or to advance crop improvement in the cultivated strawberry. We chose 7 genes from the 589 RP genes for further analysis of their promoters. The expression profiles of these seven RP genes differ in level and developmental stage (Fig. 2; Fig S1; Dataset S1). For example, genes 21624, 19774, 02647, and 03606 are expressed in the young floral receptacle at flower stages 6–7 (Floral Receptacle6_7) and their expression levels in the receptacle increase from fruit stage 1 (cortex1 and pith1) all the way to the green stage (Fig. 2; Fig S1).

In contrast, genes 25908 and 06301 are expressed in neither the young floral receptacle nor stage 1 receptacle fruit (cortex1 or pith1), both of which are pre-fertilization stages. Immediately after fertilization (stage 2 onward), their expression is detectable in the receptacle (cortex and pith) and the expression levels are highest from stage 3 through stage 5 (Fig. 2; Fig S1). We also selected genes with markedly different expression levels; genes 21624 and 19774 are expressed at levels 50–100 times greater in the receptacle (reads per kilobase million (RPKM) > 10,000 in pith post fertilization) than all other selected genes (Fig. 2; Dataset S1). However, these two genes are also expressed in other profiled tissues and are thus less tissue specific. Gene02647 is expressed more highly in cortex than in pith (Dataset S1; Fig S1). Together, this set of genes provides a range of expression characteristics that may be useful for different application purposes. Further, as *KNOTTED1-LIKE* homeobox transcription factors, genes 02647 and 03606 are putative regulators of meristem identity and potentially valuable for future applications to manipulate strawberry fruit development. Thus, we isolated 2–2.5 kb genomic sequences (‘regulatory sequences’) upstream of the ATG start codon of each of the seven genes and asked if the regulatory sequences were sufficient to drive expression of the GUS reporter in the receptacle. Regulatory sequences that activate GUS expression specifically in the receptacle will be useful to drive genes of interest in future studies.

### Expression analysis of *promoter::GUS* reporters

#### Strong promoters

**Fig. 3:**
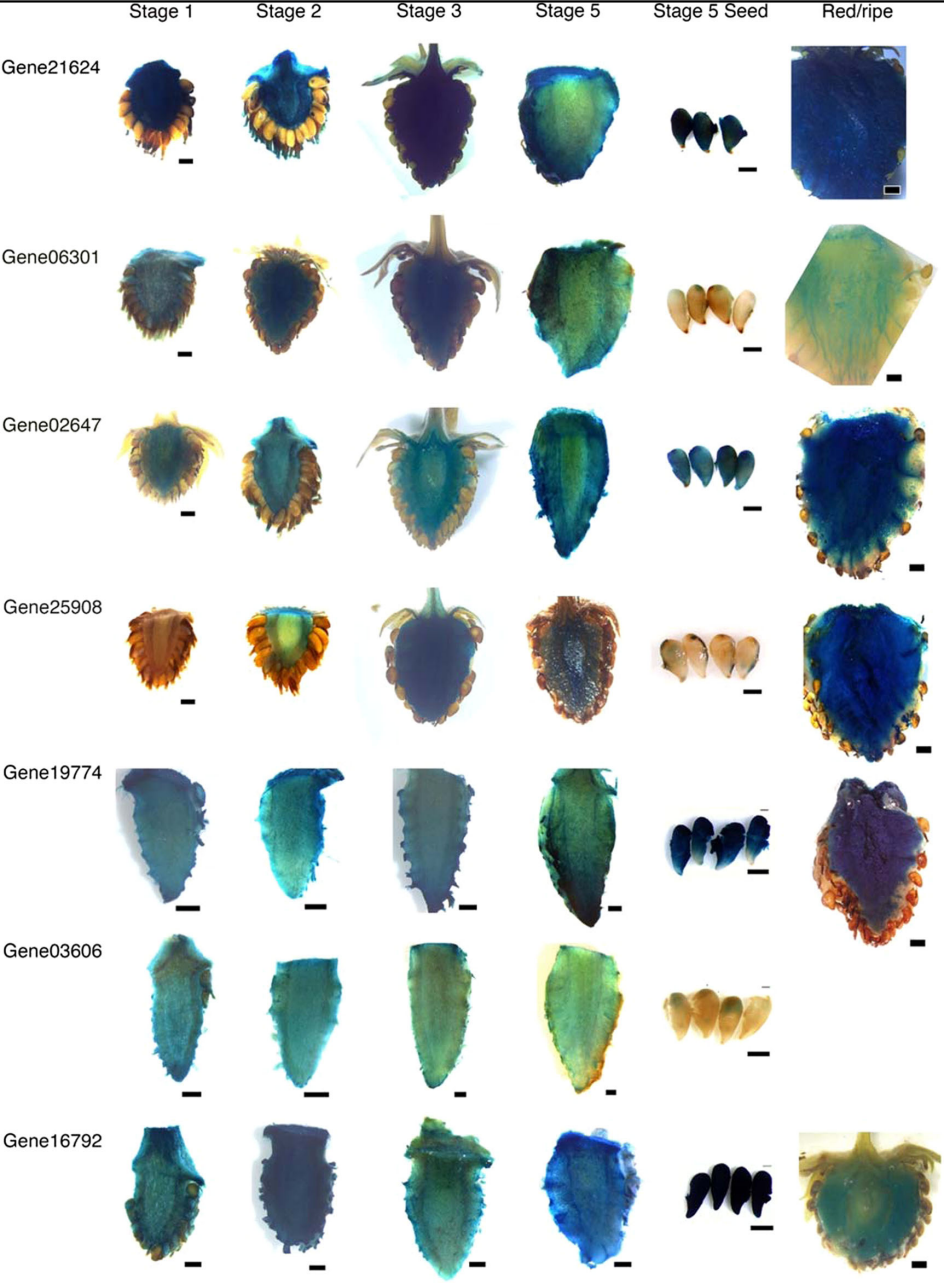
Isolated regulatory sequences drive expression of the β-glucuronidase (GUS) reporter gene in the receptacle

Consistent with the transcriptome data (Fig S1), the tested sequences successfully induced expression of the GUS reporter in the receptacle (Fig. 3), but not in leaves, petals, anthers, or sepals (Fig S2). The regulatory sequences of genes 19774 and 21624 are particularly strong and GUS staining of both receptacles and stage 5 seeds was visible after only a few minutes in the staining buffer. These two promoters will be useful when a very high, though less receptacle-specific, level of gene expression is required.

Receptacles from stage 1 to ripe and stage 5 seeds from transgenic plants expressing seven *promoter::GUS* constructs are shown by GUS staining. Scale bars for promoters of genes 21624, 06301, 02647, and 25908 are 1 mm for receptacles and 500 µm for seeds. Scale bars for promoters of genes 19774, 03606, and 16792 are 500 µm for stage 1–5 receptacles and seeds. Scale bars for ripe receptacles of promoters of genes 19774 and 16792 are 1 mm.

#### Receptacle-specific promoters

In contrast, the promoters of gene03606, a KNOX transcription factor, gene06301, a MADS-Box transcription factor, and gene25908, an LRR (leucine-rich repeat) receptor-like kinase, are active primarily in the receptacle and induce minimal GUS staining in seeds, at least at stage 5 (Fig. 3). These promoters will be ideal for driving gene expression in the fleshy fruit, but not the seed. In the receptacle, genes 03606, 06301, and 25908 are expressed in the range of 100–200 RPKM, a level which is 50–100 times lower than that of genes 21624 and 19774 (Dataset S1).

#### Fertilization-induced promoters

GUS staining is evident in post-fertilization receptacles ranging from stages 2 to 5 from plants expressing *pGene25908::GUS, pGene02647::GUS, pGene06301::GUS, pGene21624::GUS*, and *pGene16792::GUS* constructs (Fig. 3). GUS staining in receptacles expressing *pGene25908::GUS* is undetectable at stage 1, faint at stage 2, and strong from stages 3 to 5 (Fig. 3), indicating increased transcriptional activity post fertilization. The promoter of gene25908 will be uniquely useful to drive a gene of interest to be induced by fertilization, in contrast to the other four promoters which appear active pre-fertilization but provide stronger expression post fertilization.

#### Expression in ripening receptacle

Given broad interest in genetic manipulation of fruit ripening to improve color, taste, and texture, promoters that can drive gene expression in the receptacle at ripening stage are of great interest and utility. We stained ripened receptacles of transgenic plants expressing six of the seven *promoter::GUS* constructs. The promoters of genes 21624, 02647, 19774, 16792, and 25908 are active at ripe stage and are therefore potentially useful for applications in mature fruit. The promoter of gene06301, however, induced only minimal staining in the vasculature of ripe receptacles (Fig. 3).

### Two *cis*-regulatory elements are detected in the RP promoters

*Cis*-regulatory elements within promoter sequences influence the expression of driven genes to be constitutive, tissue specific, responsive to external factors, or some combination of the three^29^. Therefore, knowledge of *cis*-regulatory elements can be important when choosing promoter sequences to drive transgenes of interest. To our knowledge, there is no chromatin immunoprecipitation**-**sequencing data yet available for strawberry and no database currently hosts in silico-predicted strawberry transcription factor binding site data for *cis*-element analysis. However, the recently updated PlantPAN 3.0 database^30^ hosts in silico*-*predicted transcription factor binding sequences from apple (*Malus domestica*), a species closely related to strawberry. These data are based on homology analyses with *Arabidopsis* transcription factors for which there are experimental data to indicate binding sites. Analysis of the seven tested promoter sequences with this database identified binding sites predicted to be recognized by two apple transcription factors (Dataset S3). Consensus sequence GGTTA, found in all seven promoters, is recognized by gene MDP0000164819, which has homology to the *Arabidopsis* trihelix transcription factor GT-1. In *Arabidopsis*, GT transcription factors bind to GT regulatory elements, which are found in the promoters of light-responsive genes^31^. This result suggests that the activities of the promoters reported here could be affected by light, a consideration that should be kept in mind when using the promoters to test gene function in strawberry. Consensus sequence GCGTA, found in the promoters of genes 03606, 21624, and 19774, is recognized by gene MDP0000762302, a NAC domain-containing transcription factor. Dataset S3 provides the location of the regulatory elements in each promoter sequence. More thorough regulatory element analyses will be possible as new transcription factor data for strawberry becomes available. Future work will be necessary to test if the two identified *cis*-elements may contribute to the receptacle preferential expression.

## Discussion

In this study, we performed differential expression analyses on previously generated *F. vesca* fruit and flower RNA-Seq data to identify 589 genes that are more highly expressed in the receptacle versus in all other profiled reproductive and vegetative tissues. Isolated upstream regulatory sequences from 7 of the 589 genes successfully drive expression of the GUS reporter in the receptacle (Fig. 3) but not in leaves or flowers (Fig S2). These sequences may now be used as tissue-specific promoters to drive expression of genes of interest to study or manipulate receptacle fruit. The advantages, disadvantages, and suggested applications of each of the tested promoter sequences are summarized in Table 2.

**Table 2:** Characteristics and potential applications of tested promoter sequences

Promoter	Description	Applications
pGene19774	Strong expression in receptacle and seeds from stage 1 to ripe.	Overexpress genes of interest or drive RNAi constructs. When one desires high level of expression despite less tissue specificity. When strong expression in ripe fruit is needed.
pGene21624	Strong expression in receptacle and seeds from stage 1 to ripe.	Overexpress genes of interest or drive RNAi constructs. When one desires high level of expression despite less tissue specificity. When strong expression in ripe fruit is needed.
pGene03606	Expressed in receptacle but not seed from stages 1 to 5.	Drive receptacle-specific expression of genes of interest or RNAi constructs. When one desires preferential expression in receptacle.
pGene06301	Expressed in receptacle but not seed. From stage 1 to ripe, expression decreases as fruit matures.	Drive receptacle-specific expression of genes of interest or RNAi constructs. When one desires receptacle-specific expression at the early stages of fruit development.
pGene25908	Expressed in receptacle but not seed. Expression is induced by fertilization (from stage 2 to ripe).	Drive receptacle-specific and fertilization-induced expression of genes of interest or RNAi constructs.
pGene02647	Expressed in receptacle but also in seed from stage 1 to ripe. Expression level increases post fertilization.	Drive expression of genes of interest or RNAi construct. When one desires gradual increase of expression in receptacle and seed post fertilization (from stage 2 to ripe). Drive expression in ripe fruit.
pGene16792	Expressed in receptacle but also in seed from stage 1 to ripe. Expression increases post fertilization.	Drive expression of genes of interest or RNAi construct. When one desires gradual increase of expression in receptacle and seed post fertilization (from stage 2 to ripe). Drive expression in ripe fruit.

The identified RP genes, particularly transcription factors, are candidate regulators of receptacle fruit development and further elucidation of their functions in strawberry may provide insight into general fleshy fruit developmental processes. Among the RP genes are several homologs of *Arabidopsis* meristem regulators, e.g., MADS-Box gene *FveSVP (*gene30741) and *KNOTTED 1-LIKE* homeobox genes (genes 03606 and 02647), which is consistent with previous data suggesting that the receptacle retains meristematic identity during the early stages of fruit development^21,32^. Tissue-specific meristem regulators identified in the present study may be exploited to extend the meristematic activity of receptacle fruit during development. This opens the door for future efforts to manipulate strawberry fruit size and shape.

### Isolated regulatory sequences are useful to drive expression of genes of interest in the receptacle

Based on analysis of comprehensive RNA-Seq datasets, the work presented here provides a suite of promoters with varied levels and timing of expression specifically in strawberry fruit. This is an advancement over previous work which was conducted prior to the availability of global gene expression studies^14,16–19^. The promoter sequences reported here are useful research tools for the community and may be used for a variety of purposes including driving expression of tissue-specific reporters, RNA interference (RNAi) constructs, or specific genes to manipulate fruit development. For example, to manipulate fruit size, genes regulating meristem identity in the receptacle, e.g., *FveSVP*, or biosynthesis of hormones, e.g., auxin and GA, may be mis-expressed. In particular, genes 19774 and 21624 are extremely highly expressed in reproductive tissues and their regulatory sequences could thus drive strong constitutive expression in the receptacle. These promoters could be useful to manipulate fruit nutrition, color, flavor, or pest and pathogen defense^33–35^. The promoter of gene25908 is specifically active post fertilization in the receptacle and could be used to test the functions of candidate regulators of fruit identity. For example, MADS-Box genes such as *FveSEP1* are active throughout both flower and fruit development. To untangle a potential role in fruit identity from a role in floral identity, the promoter of gene25908 could drive RNAi to specifically down-regulate *FveSEP1* post fertilization during fruit development.

In *Arabidopsis*, promoter deletion experiments suggest that most genes have functional promoters within ~1400 bp of the translation start site^36–38^. No similar experiments have thus far been conducted in strawberry, but the *F. vesca* genome size and gene density (240 Mb and 7 kb/gene, respectively) are only slightly larger than those of *Arabidopsis* (135 Mb and 4.9 kb/gene, respectively)^3,39,40^. Therefore, in an attempt to capture the entirety of the promoter regions, we amplified and cloned a 2–2.5 kb region upstream of the ATG start codon of each of the selected RP genes. It is possible that the cloned regions do not contain full promoter sequences. However, since the goal is to identify promoters for use in future experiments, the GUS reporter should reflect the activity of the selected regulatory sequences even if the GUS expression is not identical to the endogenous promoter activity. Indeed, stage 1 *pGene06301* receptacles show moderate staining despite very low levels of gene06301 transcript detected by RNA-Seq at stage 1 (Dataset S1).

Additionally, stability of the GUS protein reflects cumulative gene expression and is therefore not directly quantitative. For example, overnight staining detects promoter activity in stage 5 seeds for genes 02647 and 16792 (Fig. 3). This staining is stronger than expected given that RPKM levels in seed are only a fraction of those in the receptacle for both genes (Dataset S1). This result could be explained by perdurance of the stable GUS protein^41^ rather than by differences in activity of the isolated regulatory sequence and the native promoter.

### MADS-Box transcription factors as candidate regulators of fruit identity

Despite detailed characterization of MADS-Box genes, including *SEP1*, in floral organ identity specification, factors that establish and maintain fruit identity are unknown. Previous studies examining the late stages of fruit development also indicated roles for MADS-Box genes in fleshy fruit ripening in multiple species, including tomato^26,42,43^, apple^44,45^, peach^46^, and the cultivated strawberry, *F. ananassa*^11^. Homologs of *FUL* have been demonstrated to promote ethylene-independent ripening processes in tomato^28^ and suppression of *SEP1/2-like* gene function reduces ripening in apple^47^. Our data show that in strawberry, *FveFUL* and *FveSEP1* are highly expressed in the receptacle throughout all profiled stages of fruit development (Dataset S1), indicating that their functions may not be limited to floral organ identity and ripening. *FveFUL* and *FveSEP1* are perhaps broadly involved in promoting fruit identity, a process which is not fully understood in *Arabidopsis*, tomato, or non-ovary-derived fleshy fruit^48^. Functional characterization will be required to determine if one or both are required for receptacle fruit identity. Subsequent comparison with studies from *Arabidopsis* and tomato will provide insight into how conserved genes contribute to general developmental processes or potentially will shed light on how a receptacle-derived fruit differs from ovary-derived fruit.

## Materials and methods

### Differential expression analyses to identify receptacle fruit-associated genes

Both DESeq2 and edgeR were used in R version 3.3.1.

#### DESeq2

Following its vignette, DESeq2 version 1.12.14 ^24^ was used to identify genes with differential expression between all profiled stages of cortex and pith tissues and all other tissues included in previously generated flower and fruit transcriptome datasets^20–22^. Read counts mapped against CDS (coding sequence) without normalization were used as input. Two groups, ‘fruit’ and ‘other tissues,’ were compared to identify differentially expressed genes. The ‘fruit’ group contained data for cortex stages 1–5 and pith stages 1–5 (two biological replicates each). The ‘other tissues’ group contained all other tissues profiled in both the flower transcriptome dataset^21^ and the early and late stage fruit transcriptome datasets^20,22^. This method does not differentiate between the stages profiled for each tissue and is therefore a conservative approach due to the resulting large dispersion estimates. The *p* value was adjusted using the Benjamini–Hochberg method. Cutoff was set at *p*adj < 0.05 and log2 fold change > 2.

#### edgeR

Following the edgeR user guide, a classic edgeR analysis^25^ was also used to identify genes differentially expressed between *F. vesca* cortex and pith tissues and all other profiled tissues in both the flower and fruit transcriptome datasets (edgeR version 3.14.0). The input and groups compared were the same as detailed for DESeq2 above. Prior to calling differential expression, library sizes were estimated using colSums. After filtering for lowly expressed genes, only genes with at least one read per million in at least three samples were kept. Functions were set at DGEList, calcNormfactors, estimateCommonDisp, estimateTagwiseDisp, exactTest, and topTags. False discovery rate is controlled by the Benjamini–Hochberg method. Cutoff was set at false discovery rate (FDR) < 0.05 and log fold change > 2.

Descriptions for genes identified via both DESeq2 and edgeR were taken from Plaza Dicots version 3.0 ^49^ (https://bioinformatics.psb.ugent.be/plaza/versions/plaza_v3_dicots/).

Heat maps were generated with Log2 transformed RPKM values using Morpheus, a web interface available from the Broad Institute (https://software.broadinstitute.org/morpheus/).

### Generation of GUS transcriptional reporter constructs and strawberry transformation

Sequences upstream of the ATG start codon of genes 03606 (2471 bp), 16792 (2298 bp), 19774 (2318 bp), 25908 (2115 bp), 21624 (2394 bp), 02647 (2459 bp), and 06301 (2490 bp) were PCR amplified from YW 5AF7 genomic DNA using Phusion polymerase (NEB, Cat. no. M0530S) and using primer sequences detailed in Table S2. The resulting fragments were cloned into PCR8/GW/TOPO using a TA cloning kit (Invitrogen, Cat. no. K250020). After confirmation by sequencing, promoters were subcloned into the binary vector pMDC162 ^50^ by Gateway LR reaction (Invitrogen, Cat. no. 11791–100). Corresponding gene numbers from a new annotation^4^ are as follows: gene03606 (FvH4_4g26090); gene16792 (FvH4_6g00660); gene19774 (FvH4_3g00920); gene25908 (FvH4_6g53170); gene21624 (FvH4_6g41500); gene02647 (FvH4_2g32400); and gene06301 (FvH4_5g08530).

Constructs were transformed into YW 5AF7 plants following a published protocol^2,51,52^. Briefly, young leaves from YW 5AF7 plants were infiltrated with GV3101 *Agrobacteria* containing the plasmid construct and co-cultured on an MS salt-based agar medium at pH 5.8, which was supplemented with 2% sucrose, 3.4 mg/L 6-benzylaminopurine, 0.3 mg/L indole-3-butyric acid, and 0.7% agar. After 3 days of co-cultivation in darkness, leaves were transferred to the same medium plus 250 mg/L timentin and 250 mg/L carbenicillin for 2 weeks. Then, leaves were moved again to the same medium plus 2 mg/L hygromycin. After 2 additional weeks, leaves were moved to the same medium plus 4 mg/L hygromycin. Leaves were subsequently moved to fresh plates containing the medium with 4 mg/L hygromycin every 2 weeks until shoots emerged. Healthy shoots were transferred to rooting media (0.5× MS salts plus 0.01 mg/L IBA, 2 mg/L hygromycin, 2% glucose, and 0.7% phytoagar, pH 5.8). After the development of roots, independent transgenic lines were moved to soil and genotyped using the following primers to amplify a portion of the GUS gene: GUS-F: 5’ ACCGTTTGTGTGAACAACGA 3’ and GUS-R: 5’ AATGCGAGGTACGGTAGGAGT 3’.

In total, 6 independent transgenic *gene03606p::GUS* lines, 17 *gene19774p*::GUS lines, 6 *gene16792p::GUS* lines, 7 *gene25908p::GUS* lines, 3 *gene21624p::GUS* lines, 15 *gene02647p::GUS lines*, and 8 *gene06301p::GUS* lines were generated. Similar GUS expression patterns were observed across all independent lines generated for each construct and representative images are shown (Fig. 3).

### GUS staining and photography

Seeds dissected out of the ovary, longitudinally bisected receptacle fruits, leaves, and open flowers from *T*_0_ generation transgenic plants were stored in 100 mM sodium phosphate buffer (pH 7.4) during the harvesting process. Next, sodium phosphate buffer was removed and GUS staining solution was added (100 mM sodium phosphate buffer, pH 7.4, 1 mg/mL X-glucuronic acid, 0.5 mM potassium ferricyanide, and 0.5 mM potassium ferrocyanide). Tissue was vacuum infiltrated for 30 min and then incubated overnight at 37 °C. Stained tissues were passed through an ethanol series (20%, 35%, 50%) followed by 30 min of incubation in FAA (50% ethanol, 5% formaldehyde, 10% acetic acid, water to volume). Tissues were stored in a final solution of 70% ethanol, observed using a stereo microscope, and photographed using a Zeiss Axiocam 105 color camera. The staining procedure was adapted based on published protocols^53,54^. At least 3 fruits from each developmental stage from each of at least 3 independent transgenic lines were GUS stained for all constructs. Representative photos are shown.

### GO term enrichment analysis

Analysis was performed using the web-based AgriGO tool^55^. GO enrichment was derived using Fisher’s exact test and a FDR cutoff < 0.05.

### *Cis*-regulatory element identification in tested promoter sequences

Analysis was performed using the ‘Multiple Promoter Analysis’ function of the PlantPAN 3.0 database^30^ (http://plantpan.itps.ncku.edu.tw/). In step 2 of the settings selection, transcription factors from *Malus domestica* were selected. In step 3, both Tandem Repeat and CpNpG promoter elements were selected. Descriptions of *Malus domestica* transcription factors were taken from the Plaza Dicots version 3.0 database^49^ (https://bioinformatics.psb.ugent.be/plaza/versions/plaza_v3_dicots/).

## Supplementary information

Supplemental Tables

Supplemental Figures

Dataset S1

Revised Dataset S2

Dataset S3

## Data Availability

All RNA-Seq data used in this article can be found in Sequence Read Archive at NCBI. The accession numbers are SRA065786, SRP035308, and SRR5155708 to SRR515515.
