# Supplementary material for: Identification of genes preferentially expressed in wild strawberry receptacle fruit and demonstration of their promoter activities
Source: Hortic Res. 2019 May 1;6:50. doi: 10.1038/s41438-019-0134-6 (PMC6491448; doi:10.1038/s41438-019-0134-6)
Supplement: Supplementary file 1 — Supplemental Tables [file 41438_2019_134_MOESM1_ESM.docx]

**Supplemental Tables**

**Table S1.** Descriptions of *F. vesca* tissues and developmental stages profiled in previously generated RNA-seq datasets (Kang et al., 2013; Hollender et al., 2014; Hawkins et al., 2016)

| **Sample** | **Description** |
| --- | --- |
| **Floral Tissues** | |
| Flower 1_4 | Whole flowers from stage 1 to stage 4 |
| Perianth 5_6 | Combined sepal and petal tissues from stage 5 or 6 flowers |
| Floral Receptacle 6_7 | Receptacle (stem tip) collected from stage 6 or stage 7 flowers |
| Style1 | Styles and stigmas from newly opened flowers (stage 1) |
| Style2 | Styles from developing fruit 2-4 days after the flower opens (stage 2 fruit) |
| Carpel 7_8 | Carpels collected from stage 7 or 8 flowers |
| Carpel9 | Carpels collected from stage 9 flowers |
| Carpel10 | Carpels collected from stage 10 flowers |
| Carpel12 | Carpels collected from stage 12 flowers |
| Anther6_7 | Anthers collected from stage 6 or stage 7 flowers |
| Anther7_8 | Anthers collected from stage 7 or 8 flowers |
| Anther9 | Anthers collected from stage 9 flowers |
| Anther10 | Anthers collected from stage 10 flowers |
| Anther11 | Anthers collected from stage 11 flowers |
| Anther12 | Anthers collected from stage 12 flowers |
| Pollen | Pollen collected from open flowers |
| Microspore_10 | Microspores from stage 10 flowers |
| **Fruit Tissues** | |
| Cortex1 | Cortex tissue collected from open flower, stage 1 of fruit development. Pre-fertilization. |
| Cortex2 | Cortex collected from developing fruit 2-4 days after the flower opens (stage 2 fruit). Post-fertilization. |
| Cortex3 | Cortex collected from developing fruit 6-7 days after the flower opens (stage 3 fruit) |
| Cortex4 | Cortex collected from developing fruit 8-10 days after the flower opens (stage 4 fruit) |
| Cortex5 | Cortex collected from developing fruit 10-13 days after the flower opens (stage 5 fruit) |
| Pith1 | Pith tissue collected from open flower, stage 1 of fruit development |
| Pith2 | Pith tissue collected from developing fruit 2-4 days after the flower opens (stage 2 fruit) |
| Pith3 | Cortex collected from developing fruit 6-7 days after the flower opens (stage 3 fruit) |
| Pith4 | Cortex collected from developing fruit 8-10 days after the flower opens (stage 4 fruit) |
| Pith5 | Cortex collected from developing fruit 10-13 days after the flower opens (stage 5 fruit) |
| Green | Receptacle tissue collected at about 15 DPA from Yellow Wonder accession of *F. vesca* |
| White | Receptacle tissue collected at about 19-21 DPA from Yellow Wonder accession of *F. vesca* |
| Ovule1 | Ovules collected from open flower. Stage 1 of fruit development |
| Seed2 | Seeds collected from developing fruit 2-4 days after the flower opens (stage 2 fruit) |
| Ghost3 | Seeds without embryos (i.e., endosperm + seed coat) from developing fruit 6-7 days after the flower opens (stage 3 fruit) |
| Ghost4 | Seeds without embryos (i.e., endosperm + seed coat) from developing fruit 8-10 days after the flower opens (stage 4 fruit) |
| Ghost5 | Seeds without embryos (i.e., endosperm + seed coat) from developing fruit 10-13 days after the flower opens (stage 5 fruit) |
| Embryo3 | Heart stage embryos collected from developing fruit 6-7 days after the flower opens (stage 3 fruit) |
| Embryo4 | Immature cotyledon stage embryos collected from developing fruit 8-10 days after the flower opens (stage 4 fruit) |
| Embryo5 | Mature embryos collected from developing fruit 10-13 days after the flower opens (stage 5 fruit) |
| Wall1 | Carpel walls from newly opened flower (stage 1 fruit) |
| Wall2 | Ovary walls from developing fruit 2-4 days after the flower opens (stage 2 fruit) |
| Wall3 | Ovary walls from developing fruit 6-7 days after the flower opens (stage 3 fruit) |
| Wall4 | Ovary walls from developing fruit 8-10 days after the flower opens (stage 4 fruit) |
| Wall5 | Ovary walls from developing fruit 10-13 days after the flower opens (stage 5 fruit) |
| **Vegetative Tissues** | |
| Leaf | Young, trifoliate leaves |
| Seedling | 10 day old, whole seedlings. Grown on MS media |

*Flower and fruit developmental stages are based on Hollender et al., 2012

**Table S2.** Primer sequences used to amplify 2 – 2.5 kb regions upstream of RP genes

| **Primer Name** | **Sequence 5’ to 3’** |
| --- | --- |
| 03606p-F | AGCCCCTGTGAAATATGAATTGC |
| 03606p-R | CATTTTGCCATGTATGTTTGCTCGG |
| 19774p-F | AGCAAGCTCACTTCCACATCCA |
| 19774p-R | CAACGCCGGCCATTTCTGAAG |
| 16792p-F | GAAACCAAGGCCAGGAGAAATG |
| 16792p-R | GATTTACTATCTAAACAGCTTCAAAGC |
| 25908p-F | CATTTCTGAACTCCAGTTACCTAC |
| 25908p-R | GCCAATGACCAGTGACTTCAACAC |
| 21624p-F | CGGTTGCGTCTCATCTACGTGAAA |
| 21624p-R | CAGCGGCCATATGGGGATTGATAG |
| 02647p-F | GCAATTGTTAGTAACATGCTCGCT |
| 02647p-R | CCTCCATTTCCAATTCAAAGACTTC |
| 06301p-F | GACGAAAATTGGTGCAGACTTCA |
| 06301p-R | CCTACCCATTTCTCTACTTCTTCTAC |
