# Supplementary material for: Identification of genes preferentially expressed in wild strawberry receptacle fruit and demonstration of their promoter activities
Source: Hortic Res. 2019 May 1;6:50. doi: 10.1038/s41438-019-0134-6 (PMC6491448; doi:10.1038/s41438-019-0134-6)
Supplement: Supplementary file 2 — Supplemental Figures [file 41438_2019_134_MOESM2_ESM.docx]

**Supplemental Figures**


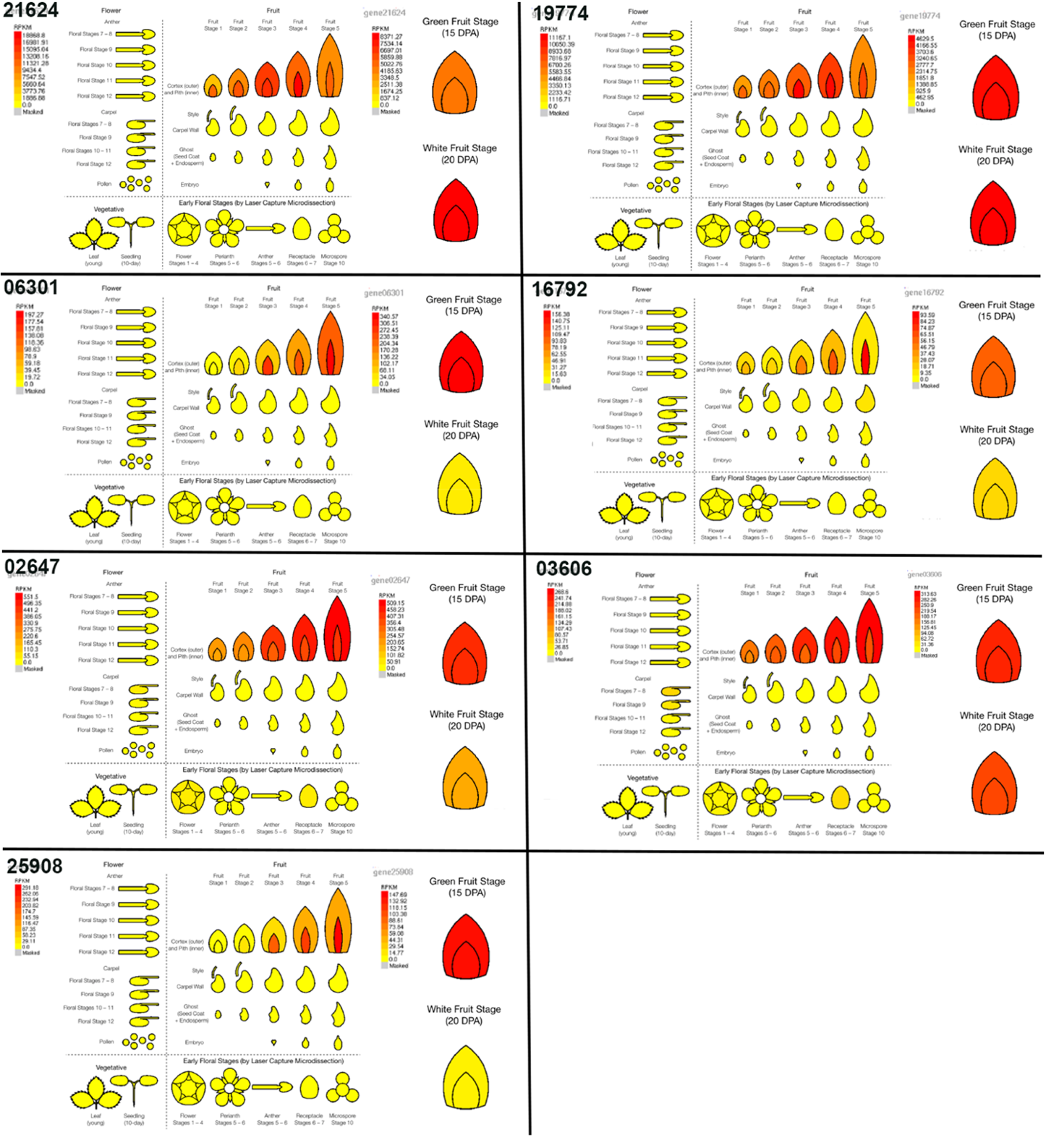


**Figure S1. eFP images showing RNA-Seq read counts in multiple tissues and stages for the seven genes selected for promoter development.** The previously generated RNA-Seq data^20-22^ show that the seven genes are all expressed during early stage fruit development (stage 1 to 5). Genes 21624, 19774, and 03606 remain highly expressed in later stage fruit (white stage). No expression is detected for any of the genes in leaf, seedling, or flower tissues.

**
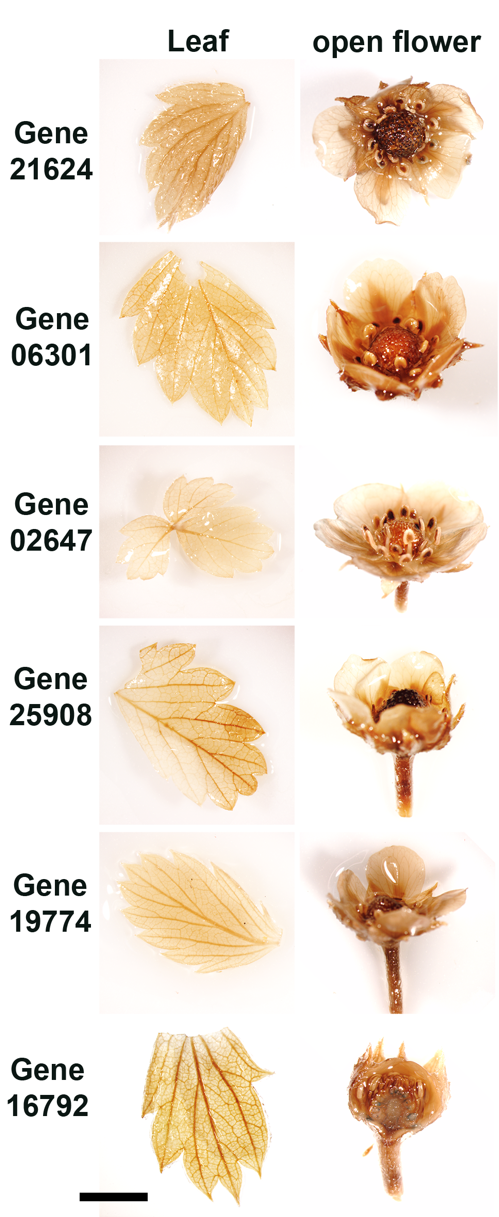
**

**Figure S2. Lack of GUS staining in leaves or the petals, stamens, or sepals of open flowers.** Tissues were harvested from plants transformed with respective *promoter::GUS* constructs. GUS staining of receptacle tissues of open flowers (stage 1) is shown in Figure 3. Scale bar is 0.5 cm.
